# Cervical metastases of squamous cell carcinoma of the maxilla: a retrospective study of 9 years

**DOI:** 10.1186/1758-3284-1-28

**Published:** 2009-07-20

**Authors:** Astrid LD Kruse, Klaus W Grätz

**Affiliations:** 1 Department of Craniomaxillofacial and Oral Surgery, University of Zurich, Switzerland

## Abstract

**Purpose:**

Metastases of squamous cell carcinoma of the tongue and the mouth floor have been well studied. Concerning maxilla squamous cell carcinomas, however, only a few studies have been performed. The question is whether a prophylactic neck dissection should be performed in these tumors.

**Patients and Material:**

In the Department of Craniomaxillofacial Surgery at the University Hospital of Zurich, 30 patients who had been treated for squamous cell carcinonoma of the maxilla were examined retrospectively. Special attention was paid to direct and late metastasis, survival rate, and treatment.

**Results:**

Of the 59 patients with upper jaw carcinomas over a 9-year period, only about half (30 patients) had a squamous cell carcinoma of the upper jaw. Of those patients, 27% had an upper lesion on the right side, 33% on the left. Of the 11 patients (36.7%) presenting positive lymph nodes, 4 patients had direct positive lymph nodes while 7 patients had later positive lymph nodes; and 71.4% of the late metastasis appeared during the first year.

**Conclusion:**

Because of the 36.7% of patients presenting metastases in the cervical lymph nodes, elective neck treatment should be considered in cases even with a negative clinical examination.

## Introduction

The estimated number of European patients with newly diagnosed cancers of the oral cavity and pharynx is 97,800 per year, and the estimated number of deaths because of these carcinomas is 40,100 in Europe per year. The worldwide incidence of oropharyngeal cancer is 8.3 cases per 100.000 [1]. Squamous cell carcinoma is the most common type of tumor among cancers in the oral cavity. These tumors metastasize, usually through the lymphatic system, to cervical lymph nodes in levels I and II [2,3]. Several studies have been done on the metastasis of squamous cell carcinoma of the tongue/mouth floor and, in particular, on prophylactic neck dissection for tongue cancer [4]. It is well documented that patients with negative cervical lymph nodes have a good prognosis, but if lymph node metastasis occurs after excision of the primary tumor, the prognosis is poor.

Only a few investigations have been done into the metastasis of squamous cell carcinoma of the upper jaw. But it is striking that the incidence of cervical lymph node metastasis from cancer of the maxilla is significant [5]. Therefore, this retrospective study will present data concerning the proportion of metastasis in squamous cell carcinomas of the upper jaw.

## Methods

Between January 1999 and July 2007, 59 patients with a malignant tumor of the upper jaw were referred to the Department of Cranio-Maxillofacial Surgery at the University Hospital of Zurich; of these, 30 patients had a squamous cell carcinoma of the upper jaw.

The female-male rate was 17:13, and the mean age of the first diagnosis was 73.1 years (in an age range of 32 – 91 years). All were staged by using the system of the International Union Against Cancer [6]. Pathohistological grading was also considered.

## Results

**Figure 1:**
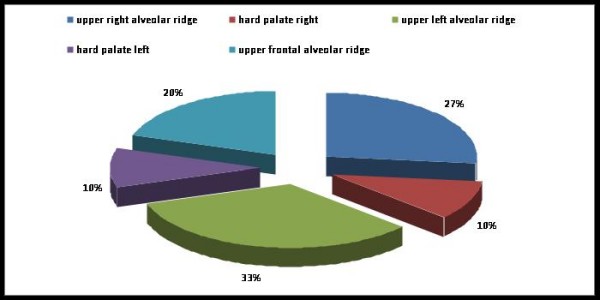
**Distribution of localizations in the upper jaw**.

A great number of the upper jaw tumors were found in the upper alveolar ridge (33% on the left, 27% on the right) (Fig. 1).

**Figure 2:**
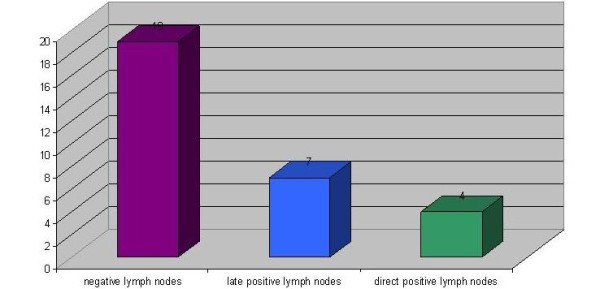
**Distribution of patients with lymph node status**.

Out of 30 patients with squamous cell carcinoma of the upper jaw, 11 patients (36.7%) presented positive lymph nodes, with direct positive lymph nodes in 4 patients and later positive lymph nodes in 7 patients (Fig. 2).

**Figure 3:**
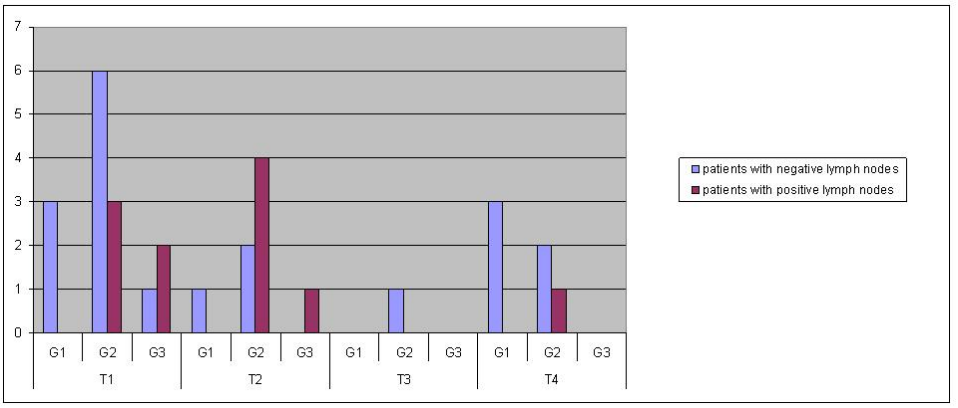
**Distribution of tumor size and grading**.

The correlation between pathological T-status and the occurrence of metastatic lymph nodes is presented in fig 3. Of patients with positive lymph nodes, 5 were found in T1-tumors and 5 in T2-tumors (Fig. 3)

Indications for neck dissection were clinical involvement of the cervical lymph nodes and suspect cervical lymph nodes as a result of a Ct, MRI, or PET/Ct scan.

A supraomohyoidal neck dissection was performed on 2 patients because of suspect lymph nodes in a CT-scan, MRI, or PET, but the pathohistological exam revealed no positive lymph nodes in these cases. However, no metastasis has been revealed in these 2 patients up to now.

**Figure 4:**
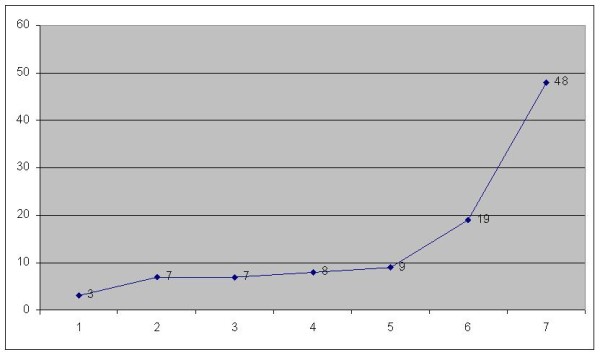
**Time (months) elapsed till appearance of late lymph node metastasis**.

The most common pattern for positive cervical lymph nodes was in the present study at T1 G2 and T2 G2 status. In cases of late metastasis, 71.4% appeared during the first year (Fig. 4).

**Figure 5:**
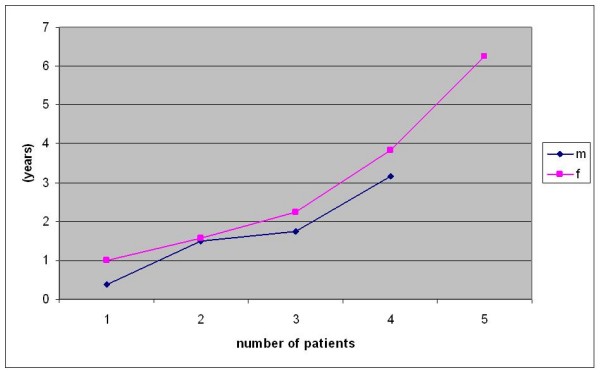
**Distribution of deceased**.

Of the 30 patients, 9 are dead of disease (Fig. 5). The 4 patients with direct positive lymph nodes are all alive, while 4 out of the 7 patients with late positive lymph nodes are deceased.

## Discussion

Clinicopathologic factors associated with the development of cervical lymph node metastasis have been well studied for other locations like tongue, mouth floor, and cheek, in particular concerning tumor size (in tongue carcinoma ≥3 mm), tumor depth (≥4 mm in tongue carcinoma), differentiation, mode of invasion, microvascular invasion, and histologic grade of malignancy [7-9]. The presence or absence of lymph node metastasis is one major prognostic factor for survival in patients with negative cervical lymph nodes [10]. A high incidence (20–30%) of cervical metastasis of cancer in the tongue/mouth floor has been well studied [7-9]. But very few studies have been performed concerning squamous cell carcinoma of the maxilla [5].

The results in the present study reveal that the higher the grading, the higher the risk of cervical metastasis. These results correlate with those of Sparamo et al [8] (2004) for tongue cancer.

Simental et al. (2006) [5] found cervical metastasis from squamous cell carcinoma of the maxillary alveolus and hard palate in 34.6% of patients. In the present study the portion of patients with cervical metastasis was similiar (33.6%).

Haddadin et al. (1998) [4], Kligerman et al. (1994) [11] and Capote et al. (2006) [12] found that patients with clinical T1/T2 tongue cancer who underwent a synchronous neck dissection had an improved survival outcome. Also O'Brien et al. (2000) [13] found occult metastatic disease in 30% of patients, and Lim et al. (2006) [14-16] found it in 28%. Therefore, regarding the proportion of late cervical metastasis, the question arises whether an elective neck dissection should be provided in early-stage squamous cell carcinoma. Capote et al. (2006) [12] reported that in pT1N0 and pT2N0 oral squamous cell carcinoma, neck dissection therapy was a significant prognostic factor for recurrence and survival. Therefore tumor size, tumor depth, and differentiation should be taken into consideration for the planning of neck dissection for squamous cell carcinoma of the upper jaw. Also the mode of invasion plays an important role in therapy planning because in certain localizations like the palate, the tumor does not need to invade very deeply before reaching the bone. In the current study, it was surprising that only 6 patients revealed a T4 status.

Another problem that should be considered seems to be the detection of micrometastasis. The assessment of the status of cervical lymph nodes is difficult, and therefore a treatment of patients with a clinical stage N0 neck is controversial. In most studies the use of CT has an error rate ranging from 7.5 to 19% [17].

In the late 1990s the positron emission tomography (PET) using F-18 fluorodecyglucose (FDG), a functional imaging methodology that provides information about tissue glucose metabolism, was applied. PET consequently a high FDG accumulation is manifested on PET images, but inflammation also reveals an increased FDG uptake and can lead to false-positive results. On the other hand, low tumor metabolic activity, the presence of small lesions, and hypoglycemia can lead to false-negative results [18]. Concerning cervical lymph nodes, Ng et al. (2005) [19] reported that sensitivity and specificity of PET images were 75% and 93%, respectively. Sigg et al. (2003) [20] reported a sensitivity of 93% and a specificity of 100%.

PET together with CT images showed a 15% increase in the accurate identification of nodal staging over using the PET images alone [21]. PET/CT seems to have a higher sensitivity and specificity for detecting lymph node metastasis [21,22]. In the last time the role of sentinel lymph node biopsies is discussed [23].

It is possible that prognostic markers – e.g., p53, CD44, cyclin D1 gene, and epidermal growth factor receptors for occult cervical lymph node metastasis that have been studied [24,25] – can help to optimize the treatment in patients with upper jaw cancer and occult metastatic lymph nodes.

The main limitation of this study was the low number of patients and the lack of carcinomas arising from the maxillary sinus. But considering the limitations of this study, multicenter studies should be performed in order to develop evidence-based protocols for treatment of squamous cell carcinomas of the maxilla.

## Conclusion

Because of the 36.7% of the patients presenting metastases in the cervical lymph nodes from squamous cell carcinoma of the upper jaw, elective neck treatment should be considered even in cases with a negative clinical examination.

## Competing interests

The authors declare that they have no competing interests.

## Authors' contributions

AK carried out the analysis of the patients' data and KWG participated in the design of the study and coordination.
